# Why Depressed Mood is Adaptive: A Numerical Proof of Principle for an Evolutionary Systems Theory of Depression

**DOI:** 10.5334/cpsy.70

**Published:** 2021-06-02

**Authors:** Axel Constant, Casper Hesp, Christopher G. Davey, Karl J. Friston, Paul B. Badcock

**Affiliations:** 1 Charles Perkins Centre, The University of Sydney, AU; 2 Culture, Mind, and Brain Program, McGill University, CA; 3 Wellcome Trust Centre for Human Neuroimaging, University College London, UK; 4 Department of Developmental Psychology, University of Amsterdam, NL; 5 Amsterdam Brain and Cognition Center, University of Amsterdam, NL; 6 Institute for Advanced Study, University of Amsterdam, NL; 7 Centre for Youth Mental Health, The University of Melbourne, AU; 8 Department of Psychiatry, The University of Melbourne, AU; 9 Orygen, AU

**Keywords:** Depression, Anhedonia, Social withdrawal, Active inference, Adaptive Prior, Computational phenotyping, Evolutionary systems theory, Simulation study, Social risk hypothesis, Two-arm bandit

## Abstract

We provide a proof of principle for an evolutionary systems theory (EST) of depression. This theory suggests that normative depressive symptoms counter socioenvironmental volatility by increasing interpersonal support via social signalling and that this response depends upon the encoding of uncertainty about social contingencies, which can be targeted by neuromodulatory antidepressants. We simulated agents that committed to a series of decisions in a social two-arm bandit task before and after social adversity, which precipitated depressive symptoms. Responses to social adversity were modelled under various combinations of social support and pharmacotherapy. The normative depressive phenotype responded positively to social support and simulated treatments with antidepressants. Attracting social support and administering antidepressants also alleviated anhedonia and social withdrawal, speaking to improvements in interpersonal relationships. These results support the EST of depression by demonstrating that following adversity, normative depressed mood preserved social inclusion with appropriate interpersonal support or pharmacotherapy.

## Introduction

It has recently been proposed that depressed mood reflects an adaptive, socially risk-averse psychobiological strategy that preserves social relationships (i.e., inclusion) when there is evidence for maladaptive instability in interpersonal exchanges (Badcock et al., 2017). This perspective follows an evolutionary systems theory (EST) of human biobehaviour called the hierarchically mechanistic mind, which combines insights drawn from research in psychology with the computational resources borrowed from the theory of active inference in theoretical neurobiology (Badcock, Friston, & Ramstead, 2019). This model rests on two fundamental claims. The first conforms to the theory of active inference by suggesting that the brain comprises hierarchically organized neurocognitive mechanisms that reduce the dispersion or decay of our sensory and phenotypic states—by generating action-perception cycles that minimize surprising exchanges with the world. The second claim ensues from an embodied perspective on neural form and function—that accommodates the broader evolutionary, developmental, and real-time processes that act on human phenotypes. The implication here is that to understand a phenotypic trait, we need approaches that synthesise findings from diverse fields of inquiry to explain both why that trait is adaptive, along with how it emerges from the nested dynamics across different timescales. In this spirit, the current study provides proof of principle for the EST of depression, using simulations of active inference. We conclude by discussing the clinical implications of our model.

Our proof of principle integrates two major schools of thought. The first is rooted in evolutionary psychological approaches to depression, rallied around the social risk hypothesis (SRH) proposed by Allen and Badcock (Allen & Badcock, 2003). Psychological symptoms of depression include feelings of sadness, emptiness, and hopelessness, along with systematic disinterest in activities (i.e., anhedonia), feelings of worthlessness, and inappropriate guilt. Typically, a diagnosis of depression is made when symptoms have been present for at least 14 days (American Psychiatric Association, 2013). Two important symptoms of depression are anhedonia and social withdrawal: the latter is commonly observed in depression as a clinical correlate of anhedonia, but is not a formal criterion (Buckner et al., 2008). Evolutionary models of depression explain the maintenance of genetic vulnerabilities to depressive symptomatology in terms of the selective advantage of these vulnerabilities in ancestral environments (R. M. Nesse, 1990). The adaptive properties of depression are thought to be restricted to the relatively transient, normative depressed mood states that we all experience from time to time, while more severe manifestations, like those observed in major depressive disorder, reflect a dysregulation of our species-typical capacity for mood variation (Nettle, 2004). The SRH suggests that depressive symptoms might have been selected as a strategy that prevents the deterioration of interpersonal relationships. Low mood reduces one’s propensity for social risk-taking, and increases implicit signalling for social support, which reduces competitive encounters (see ***Box 1*** for background). Clinical depression occurs when this sequence becomes maladaptive; specifically, when it does not lead to a resumption of normal mood. This may be due to neurobiological or psychological deficits that maintain increased sensitivity to social instability, or to instabilities or the absence of support in the proximal environment towards which the depressed individual reacts. The increased sensitivity to social instability constitutes the basis of the neurocognitive processes leading to depressed mood, upon which selection can act (for a review see (Badcock, Friston, Ramstead, et al., 2019)).

**Box 1 Evolution and depression.**

There are three general classes of evolutionary models of normative depressed mood. The first of these – resource conservation views – claims that depressive symptoms, such as learned helplessness, are a response to a low positive reward rate and insufficient control over reward and punishment (Randolph M. Nesse, 2000), and unobtainable incentives or goals (Klinger, 1975). Low appetitive functions (e.g., anhedonia) allow the individual to fine-tune resource allocation by precluding investment in poor pay-off activities. The second class refers to the social competition model, which claims that social status (e.g., rank and position in the social group) positively correlates with access to resources that enhance reproductive success. Depressive symptoms such as social withdrawal remove the individual from conflicts and other status-impairing situations that would negatively impact their social rank (Gilbert, 1997; Price, 1967). The third, attachment model claims that given the delayed maturation of human infants (Hrdy, 2011), offspring survival necessitates intensive parental and alloparental investment. Behaviours designed to maintain proximity to caregivers are instigated when significant affectional bonds are threatened. In the face of precarious interpersonal relationships, depressive symptoms should promote help-seeking and inhibit exploratory behavior and risk-taking, thereby maintaining relationships with the proximal familial environment, while avoiding the deterioration of current social bonds (Ingram et al., 1998). Standing alone, it has been argued that these models cannot account for the full scope of the depressive phenotype (Allen & Badcock, 2006). Darwinian models do not provide an explanation of the underlying mechanisms upon which selection can act. For instance, how is ‘resource reallocation’, ‘preventive withdrawal from the social environment’, or ‘familial bond strengthening’ implemented mechanistically? This is a problem, since the unit of selection is never a complex behavioral trait, but rather some (epi)genetic dispositions to express such traits. The Social Risk Hypothesis (SRH) was proposed to provide a solution to these issues and has since been developed into a neurobiologically plausible and empirically tractable mechanistic explanation for depressive phenomena (Badcock et al., 2017). According to this view, normative levels of depressed mood reduce the probability of deleterious social outcomes via three broad classes of action: (1) depression increases individuals’ cognitive sensitivity to environmental cues of social risk or instability; (2) it reduces their behavioural propensity for social risk-taking; and (3) it generates signalling behaviours (e.g., reassurance seeking, crying, gaze aversion) that attract social support and defuse aggressive or competitive encounters.

The second root of our simulation centres upon formal, testable models of depressive phenomena that borrow from the principles of computational psychiatry (Huys, Guitart-Masip, et al., 2015). Accordingly, a key aspect of the work reported in this paper is the attempt to model social inference – as it relates to the phenomenology of depression – from first principles. This is challenging, because of the many aetiological factors that underwrite the psychopathology and pathophysiology of depression. We try to formalise the normative aspects of depression as a Bayes optimal response to inference in the prosocial world, while considering both social and pharmacological interventions. To our knowledge, this is the first modelling work that addresses the interaction between social factors and pharmacotherapy within the same formalism. In this sense, the simulations reported here also provide a proof of principle for a model of the effects of drug treatment on neuronal computations that underlie belief updating and behaviour in depression. In brief, we make three basic assumptions that allow us to characterise the effect of drug treatment on social inference and subsequent behaviour. First, both inference and learning conform to the same (ideal Bayesian observer) principles of active inference; namely, belief updating and experience-dependent plasticity both optimise a variational free energy bound on (log) model evidence or marginal likelihood (Friston et al., 2016). Second, pharmacotherapy motivates neuromodulatory effects that, computationally, change the precision of sub-personal probabilistic beliefs (i.e., prior beliefs about states of affairs in the world or likelihood mappings between causes and consequences) (Parr et al., 2018). Finally, one cannot ignore the reciprocal coupling between an agent and her (prosocial) environment when modelling interpersonal exchanges. This requires an explicit consideration of how environmental (prosocial) contingencies respond to an agent’s behaviour (Badcock et al., 2017) (see ***Box 2*** for background). Here, the embedded aspect of interventions on the social environment (Bruineberg et al., 2018; Constant et al., 2018) was modelled by an increase in social reliability following patterns of behaviour that can be construed as social signalling. Our hope was to show that functional responses to social adversity use the same inferential mechanisms seen in pathological depression – and that psychopathology can be remediated by a combination of social support and drug therapy.

**Box 2 Computation, inference and depression.**

Computational phenotyping is a method in computational psychiatry to test hypotheses about the neurophysiology of mental disorders to inform nosology and suggest treatment approaches (Corlett & Fletcher, 2014). A computational phenotype refers to the set of measurable features of an agent; often described in terms of the ‘priors’ and ‘likelihood mappings’ of a generative model used by subjects for perception and decision-making (Schwartenbeck & Friston, 2016). The associated parameters of generative models show variation across the population upon which selection acts (Montague et al., 2012). This formal approach to phenotyping effectively reduces the phenotype to some formal priors – or prior beliefs – that personalise a (generative) model that people use to predict and interact with their (physical, physiological, prosocial or cultural) econiche. These interactions are usually cast in terms of (Bayesian) belief updating under a generative model that characterises a given phenotype. Computationally, prior probability distributions can take numerous forms (e.g., normal, Dirichlet, delta). This form depends on the state space being modelled (e.g., discrete, as in this paper, or continuous). Priors can also have different ‘temporal’ scales, relative to the scale at which (Bayesian) belief updating unfolds. ‘Adaptive priors’ are sculpted by evolutionary processes and become encoded over the course of ontogeny in the physiology and functional architectures of the brain; that is, they emerge from interactions between priors that are ‘empirical’ and ‘evolutionary’. Empirical priors are learned over development (e.g., learnt distribution of food patches), while evolutionary priors function as initial conditions that shape the learning of empirical priors (e.g., a prior preference for energy-rich food) (Friston, 2010). Technically, empirical priors arise whenever there is a hierarchical generative model. Empirical priors are the constraints offered to a lower level, from a higher level. When hierarchical models are inverted, empirical priors become informed by (empirical) data. Simulating belief updating – under a generative model – allows researchers to produce synthetic, *in silico* measurements (e.g., psychophysical and physiological responses) of the sort that are usually studied in real-world empirical contexts. The generative model can be manipulated by inducing artificial lesions in likelihood mappings, or by simulating pharmacological treatment that generally changes the priors (Parr et al., 2018). In so doing, one can generate artificial data in the context of a task that can be used in empirical studies. One can then test hypotheses by comparing artificial responses with participants’ empirical data (Cullen et al., 2018). The symptoms of depression are thought to relate to deficits impacting long-term reward evaluation through the acquisition of ‘pessimistic’ priors that entail negatively biased learning of environmental states (Huys, Daw, et al., 2015). Priors should reflect unambiguous beliefs about the world, as well as beliefs about the relation between environmental states and observed outcomes. For instance, we cannot directly infer the mood of another solely from the sensory impressions of that person’s facial expression. Rather, we must consider some prior assumptions about the person’s behavior and outcome probability over time (e.g., she usually smiles a lot, but she is not smiling today, so it is likely that something is awry). These are empirical priors. In depression, empirical priors biasing decision-making tip the balance towards pessimistic inference, thereby leading to systematically pessimist thoughts. For instance, depressive patients form negative sentences more quickly and frequently than non-depressed controls, when presented with both optimistic and pessimistic options (e.g., in the scramble sentence test) (Hindash & Amir, 2012; Rude et al., 2003). The simulations offered in this paper try to capture this belief-based phenomenology by using synthetic agents and active inference – a generic framework for (active) Bayesian inference and planning.

Our numerical proof of principle is based on active inference for discrete states, using (Markovian) generative models (Friston, Parr, et al., 2017) – see method section. We present a series of simulations based on an augmented version of a two-armed bandit game from economics, in which the agent has to choose between a risky or safe social engagement (Schwartenbeck et al., 2019). The augmentation involved offering the agent a cue option that indicates whether the risky arm is low-risk or high-risk (i.e., it indicates the social context); this contextual state alternates every other trial. Narratively, the cue corresponds to social media that provides information or evidence that reduces uncertainty about the prevailing social context. This means that healthy agents will systematically sample the cue to make an informed decision. As they sample their social environment, agents will learn the probability of reward afforded by choosing one of the two arms. Narratively, this relates to checking people’s availability before choosing among social options.

Based on the learning process characteristic of active inference, agents exposed to social adversity (e.g., rejection by social partners) will learn the likelihood of being rejected. We simulate different phenotypes, with and without social support and pharmacotherapy, which reshape the agent’s (pessimistic) prior beliefs. Our numerical analyses speak to how pharmacotherapy and social support – triggered by social signalling on social media – allows the agent to regain a normal mood. Depending on the type of intervention (social, pharmaceutical, or the lack thereof), the agent typically experiences a phase of low mood, and either spirals into persistent depression (anhedonia and social withdrawal), or returns to various levels of normal functioning. We will quantify the responses of our synthetic agent in terms of task performance and associated synthetic mood (i.e., expected reward under a given action policy) and behaviour (action selection).

**Figure 1:**
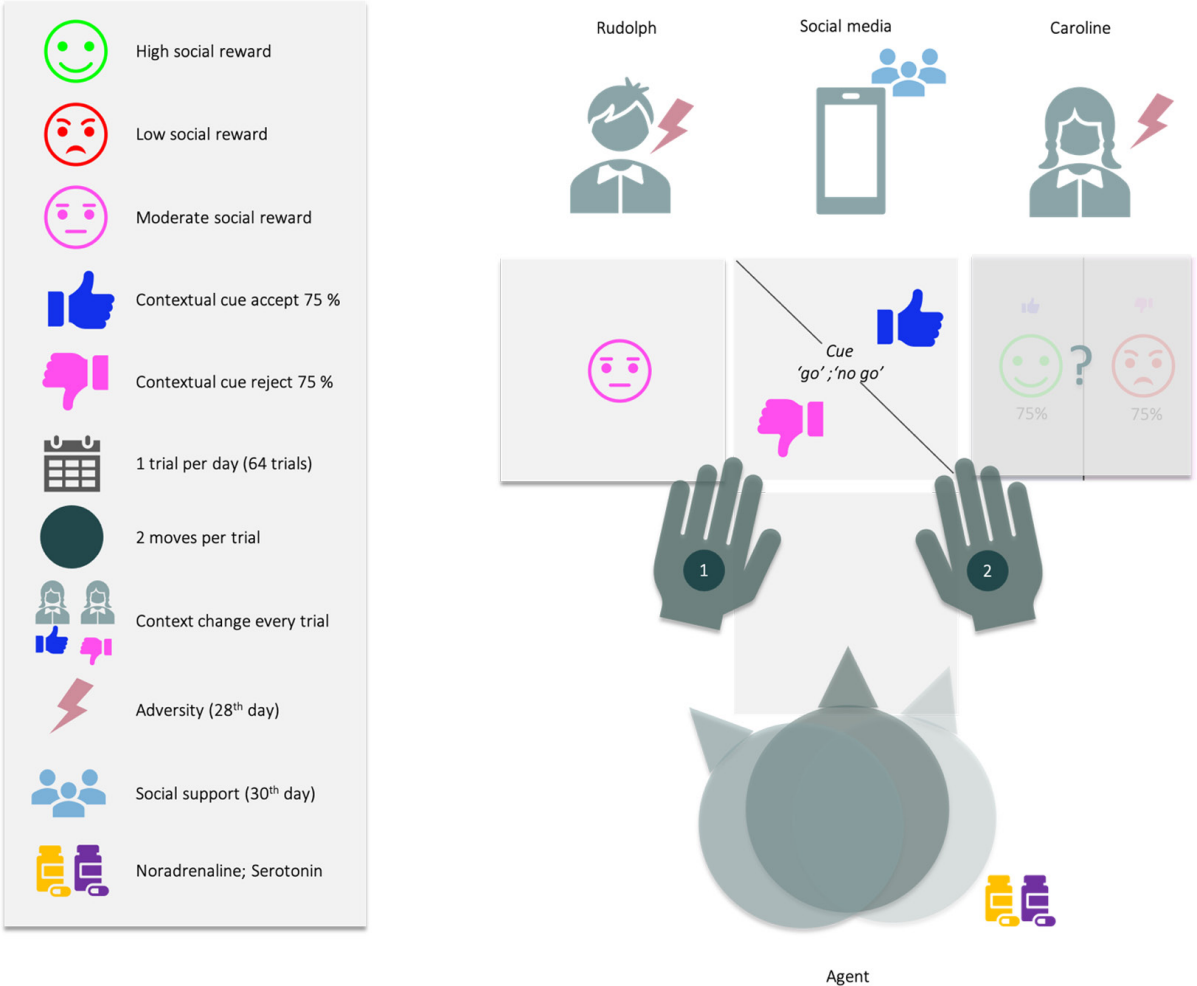
**Narrative description of the social decision-making task.** Over 64 days, the challenge is to maximise social encounters with Caroline. The agent has two moves (Caroline and Rudolph are both absorbing states, meaning that once the agent reaches them, it must stay there). For instance, on the first move, the agent can solicit information about Caroline’s availability by going on social media, and then, on the second move, decide where to go.

Our two-arm bandit social decision-making task (see ***Figure 1***) involves choosing among three social engagement options, which vary in their risk. The first is a ‘safe’ option, but with low social preference (going to a well-known friend, Rudolph, who you know can be engaged with 100% success, but won’t provide the most fulfilling interaction). The second, ‘risky’ option has a high preference (going to see a new popular student, Caroline, whom you do not know, but were told is a lot of fun), but is risky because Caroline often forfeits, and the agent is averse to failed social encounters. On a ‘good day’ the agent has a 75% chance of successfully engaging Caroline, but on a ‘busy day’, only a 25% chance of catching her. The third, ‘socially epistemic’ option yields a null cost: under this option, the agent can turn to social media, to see if Caroline is having a ‘busy day’. To characterise prosocial and emotional inference that might underwrite depression, we considered belief updating and subsequent behavior under 8 different conditions – in a three-way factorial design involving the following factors: social adversity, social support, and pharmacotherapy (see ***Table 1***).

**Table 1:** Interventions.


**Baseline**	The agent performs the social decision-making task in the absence of any adversity over a period of 64 days.

**Severe depression**	We induce social adversity on the 28^th^ day by changing the uncertainty of social outcomes. The agent is now rejected by Rudolph (always) and by Caroline on a ‘bad day’. On a good day, the odds are inverted, such that Caroline is likely to afford a negative outcome. In other words, there is a flip in contingencies of the social environment.

**Social support**	We introduce social support on the 30^th^ day, which reduces uncertainty about the outcomes of social encounters – and therefore resolves social adversity. This is modelled as an increase in Rudolph and Caroline’s reliability, which is increased when the agent forages for information on social media. Narratively, this could be interpreted as the agent signaling (implicitly or explicitly) to Caroline and Rudolph that they should be more consistent. Recovery thus depends on the sensitivity of the social environment and on how often the agent consults social media.

**Pharmacotherapy**	First-line pharmacotherapy typically employs either selective serotonin or norepinephrine reuptake inhibitors, and sometimes mixed serotonin or norepinephrine reuptake inhibitors (e.g., venlafaxine and duloxetine); the latter usually being used in patients who do not respond to serotonin reuptake inhibitors (Harmer et al., 2017). We simulate two types of synthetic pharmacotherapy: one motivated by serotonin and the other by norepinephrine. We assume, based on (Harmer et al., 2017), that serotonin upregulates prior expectations about initial states (i.e., increases the perceived probability of Caroline showing up), whereas noradrenaline introduces uncertainty about state transitions. Noradrenaline entails an overall loss of precise belief-updating during planning, a loss which underwrites the exploration of states that may lead to social reward. Condition 3 involves both noradrenaline and serotonin, condition 4 noradrenaline only, and condition 5 serotonin only.

**Social support and pharmacotherapies**	Condition 6 involves social support and both antidepressants; condition 7 involves support and noradrenaline only; and condition 8, support and serotonin only.


## Methods and materials

Active inference is a Bayesian framework that only uses local information (i.e., there is no external supervision) for belief-updating, in order to ensure biological plausibility. Markov Decision Processes (MDPs) can be used to simulate how agents infer which discrete hidden states (*s*) of the world provide the best explanation of observed sensory outcomes (*o*), under a given generative model. To generate predictions of sensory outcomes, an agent needs prior expectations about initial hidden states (an initial state prior, **D**), how states generate sensory outcomes (sensory mapping, **A**), and how states evolve over time (state transitions, **B**). The agent can infer states of the world by minimising the discrepancy between predicted and observed outcomes (a.k.a., variational free energy), or equivalently, by maximising Bayesian model evidence. For mathematical details, see (Parr & Friston, 2017).

**Figure 2:**
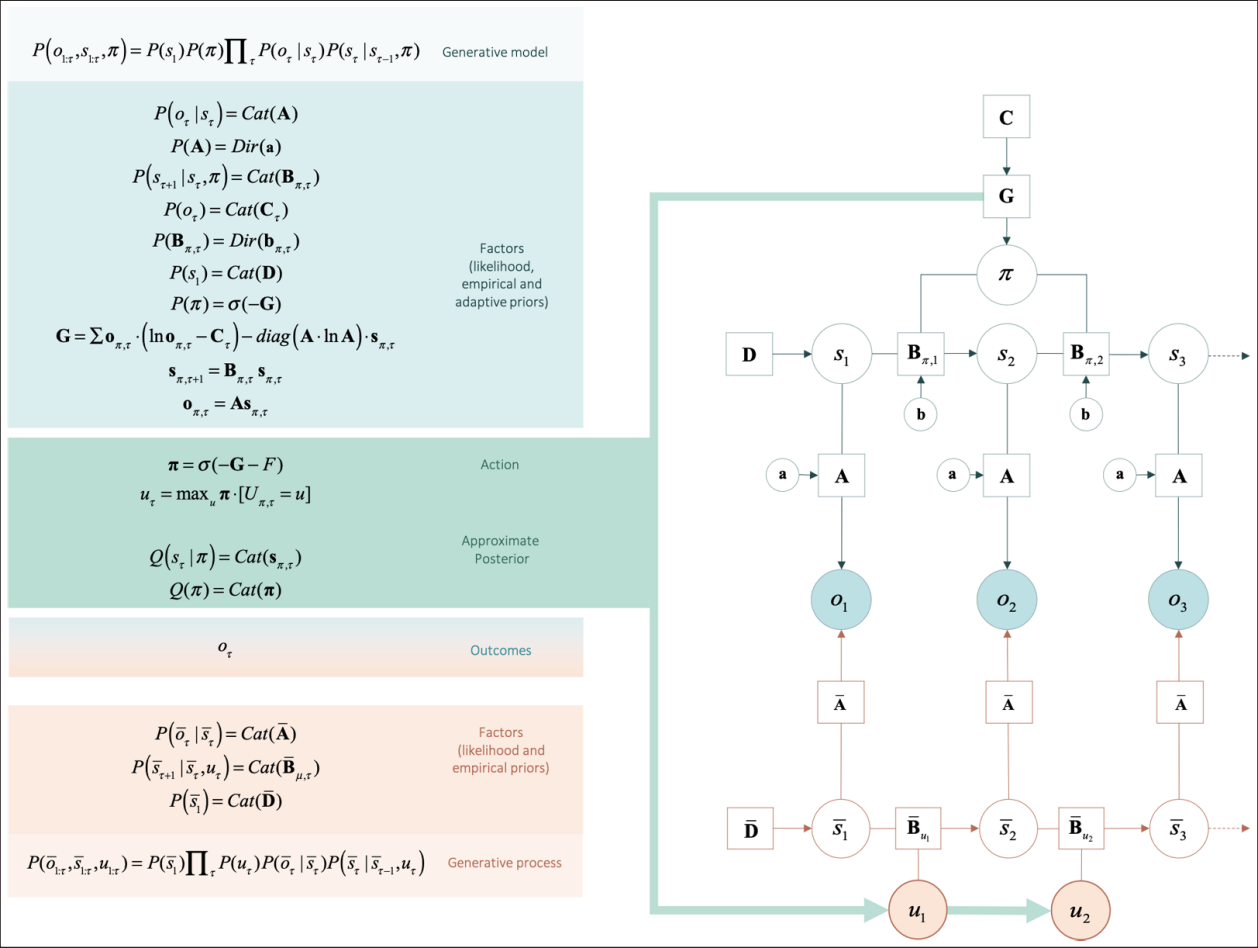
**Computational description of the decision-making task.** The generative model and generative process of our decision-making task. Open circles represent random variables (hidden states and policies), filled circles represent the outcomes, squares represent model parameters (e.g., likelihood **A**, empirical priors **B, D, G**, and the evolutionary prior **C**). The generative model is shown in the upper part of the figure, while the process generating outcomes is shown in the lower part. The generative model and process are coupled through the same outcomes (*o*) and actions (*u*), where outcomes are used to infer hidden states and policies – and action is sampled from policies to change the states that are being inferred. States of the generative model are denoted by ‘s’ while states of the generative process are denoted as ‘s_bar’. The generative model is a joint probability distribution over outcomes and hidden states, which can be decomposed into factors. Factors are conditional densities (categorical: *Cat*; or Dirichlet: *Dir*) that make up the priors and likelihood of the generative model. Priors that depend on random variables, such as hidden states and policies, are empirical priors (e.g., priors that are learnt at a given hierarchical level or time scale). Priors that do not vary on this time scale are initialised as evolutionary priors (e.g., **C**). These are log preference vectors that rank the desirability of associated outcomes. Lower-case **a** and **b** correspond to matrices of concentration parameters for **A** and **B** respectively. The process whereby outcomes are generated decomposes into a series of belief updates: (i) Policy selection: the sequence of actions (i.e., plan or policy) is inferred under prior beliefs that the most likely policy minimises expected free energy (**G**); (ii) Inference about future states depends on state transitions encoded by the transition matrix (**B**) and the likelihood (**A**); (iii) Inference about outcome: the policy – with respect to the probability transitions – generates probabilistic outcomes at each time point. The likelihood of each outcome is encoded in the likelihood matrix (**A**), which attributes the probability of each possible outcome to each possible state; and (iv) Action: the agent selects the most likely action under posterior beliefs about policies. The green arrow highlights the circular causality that results when the generative model and process are coupled through outcomes and ensuing action. The process generating outcomes triggers the message-passing, under the generative model, which entails the evaluation of a policy, from which actions are selected. Actions change states in the generative process and a new outcome is generated. Thus, the cycle of perception and action continues. Learning corresponds to updating the concentration parameters that underwrite posterior beliefs about the likelihood of the sensory matrix (**A**). Each exchange with the environment is accumulated by concentration parameters. This accumulation encodes the probability of outcomes, given hidden states – enabling the agent to learn about environmental contingencies (and the social environment to change in response to the agent’s actions). The generative model and process can be defined for any scenario. The icons in the upper panel refer to changes in the generative model induced by (simulated) pharmacotherapy, or by changes in the generative process afforded by social adversity and support. These changes are described in the next figure. For a detailed description of the update equations and underlying theory, see (Friston, Parr, et al., 2017).

When expectations of hidden states are conditioned upon the agent’s plan or policy (as encoded in the policy dependent **B** matrices), one has a generative model of action (see ***Figure 2***). Without an external referee to say what is right or wrong, the agent will need to: (i) predict her course of action, based on the succession of states, expected under each policy; and (ii) select her action based on (posterior) beliefs about the best policy. To that end, we equip the agent with (self-referential) prior beliefs that are biased towards policies with stronger expected model evidence or, equivalently, lower expected free energy, **G**.

Mathematically, expected free energy can be decomposed into pragmatic and epistemic components for any given policy. On the one hand, pragmatic value (i.e., exploitation) biases policy selection towards obtaining preferred sensory outcomes (evolutionary prior preferences, **C**), much like utility in reinforcement learning. On the other hand, epistemic value (i.e., exploration) biases policy selection towards the (expected) minimisation of uncertainty about states of the world (a.k.a., artificial curiosity).

Uncertainty can be over beliefs about current hidden states or model parameters (as quantified in free energy **F**) or over beliefs about future hidden states and their associated outcomes under a given policy (as quantified in expected free energy **G**_π_). In active inference, the latter guides action selection and can be decomposed in three distinct sources of uncertainty: (i) expected ambiguity, or anticipated uncertainty about hidden states (e.g., “how certain will I be about Caroline’s mood, given that I check social media?”), (ii) expected risk, or the anticipated uncertainty about whether future outcomes will align with preferences **C** (e.g., “how certain will I be that I obtain preferred outcomes, given that I visit Caroline?”), and (iii) the anticipated uncertainty about Dirichlet parameters of the likelihood mapping **A** (e.g., “how much might I learn about state-outcome mappings if I visit Rudolf?”) (Kaplan & Friston, 2018). Each of these sources of uncertainty can be manipulated directly with interventions on the model. Here, we focus on direct intervention on salience—via serotonergic and noradrenergic manipulation of initial states and state transitions—and on the indirect manipulation of extrinsic value via the manipulation of social observations, or outcomes (***Figure 2***). Thus, in our simulations, our agent will have a double incentive for social engagement: (i) fulfilling preferences for positive social outcomes; and (ii) the natural drive towards resolving her uncertainty over the various beliefs she has about the social world (c.f., curiosity about a new acquaintance). Crucially, it is this double incentive that we exploit to formalize the behavioural dynamics envisaged by the EST of depression; the first incentive relating to ‘evolutionary’ prior preferences for high social reward, and the second incentive relating to ‘developmental’ learning.

We limit the notion of social engagement to face-to-face encounters with Rudolph or Caroline.

The software to simulate belief updating and action selection, based on the specification of any generative model (as the one specified in ***Figure 2***), is freely available as part of the academic software SPM; specifically, the Matlab routine spm_MDP_VB_X.m (*https://www.fil.ion.ucl.ac.uk/spm/software/spm12/*).

The generative model and process used to simulate social inference – and ensuing changes in depressed mood – are described formally in ***Figure 2***. In brief, this setup considers 5 (observable) outcomes: an outcome that sets the scene for a social choice (e.g., being at home), three levels of social reward (low, moderate and high), and an epistemic cue that reports the current context (this is a ‘Go’ or ‘no-Go’ social context) that determines Caroline’s availability.

Outcomes are generated by two kinds of external states called hidden factors. The first is the context with the two levels pertaining to Caroline’s availability. These hidden states are not under the agent’s control. Conversely, transitions among the states of the second factor reflect the agents choice or policy, with four levels; i.e., home, Rudolph, Caroline, social media. The two factors interact to generate outcomes. Specifically, the context (Caroline’s availability) determines whether the social media state generates an (epistemic) outcome that is ‘go’ or ‘no-go’. Put simply, this means the agent can choose to find out whether Caroline is available or not—or contact her directly—or not. The context alternates every other day, meaning that the context-sensitive outcome available to the agent changes every other day.

Given some observations, the agent can predict outcomes under a set of plans or policies, given her beliefs about (policy-dependent) transitions among different states. This enables her to evaluate the expected free energy of each policy – and use the expected free energy as prior beliefs to form posterior beliefs, given what she has already observed. An action is generated by selecting the most likely action from the resulting posterior. And so, the cycle of perception and action continues. Notice that the coupling between the agent and the world is mediated by observable outcomes and action. The interventions corresponding to the conditions above can be modelled, either by changing the prior beliefs of the agent (about initial conditions, likelihoods or state transitions), or by changing the prosocial world in a way that responds to her choices.

## Results

We used belief updating to simulate perception, action, and learning under different levels of social adversity, support and antidepressant treatment (i.e., pharmacologically induced changes in prior beliefs about states and contingencies). The results of these simulations are summarised in ***Figures 4, 5, 6***. Behavioural outcomes and choices were assessed using the criteria listed in ***Table 2***. In what follows, we described the responses to different scenarios or conditions in turn.

**Table 2:** Synthetic diagnostic criteria.


Symptoms of normative depression	Anhedonia	Intensity	When the expected utility or reward is below the 95% confidence interval of the (healthy) control condition. Our subject experiences a lack of pleasure and disinterest in (prosocial) activities – of an intensity that a healthy phenotype experiences only about once every 20 days.

		Duration	When the intensity criterion is met for multiple consecutive trials. Narratively, the subject experiences a lack of pleasure and disinterest in (social) activities – lasting many days.

**Figure 3:**
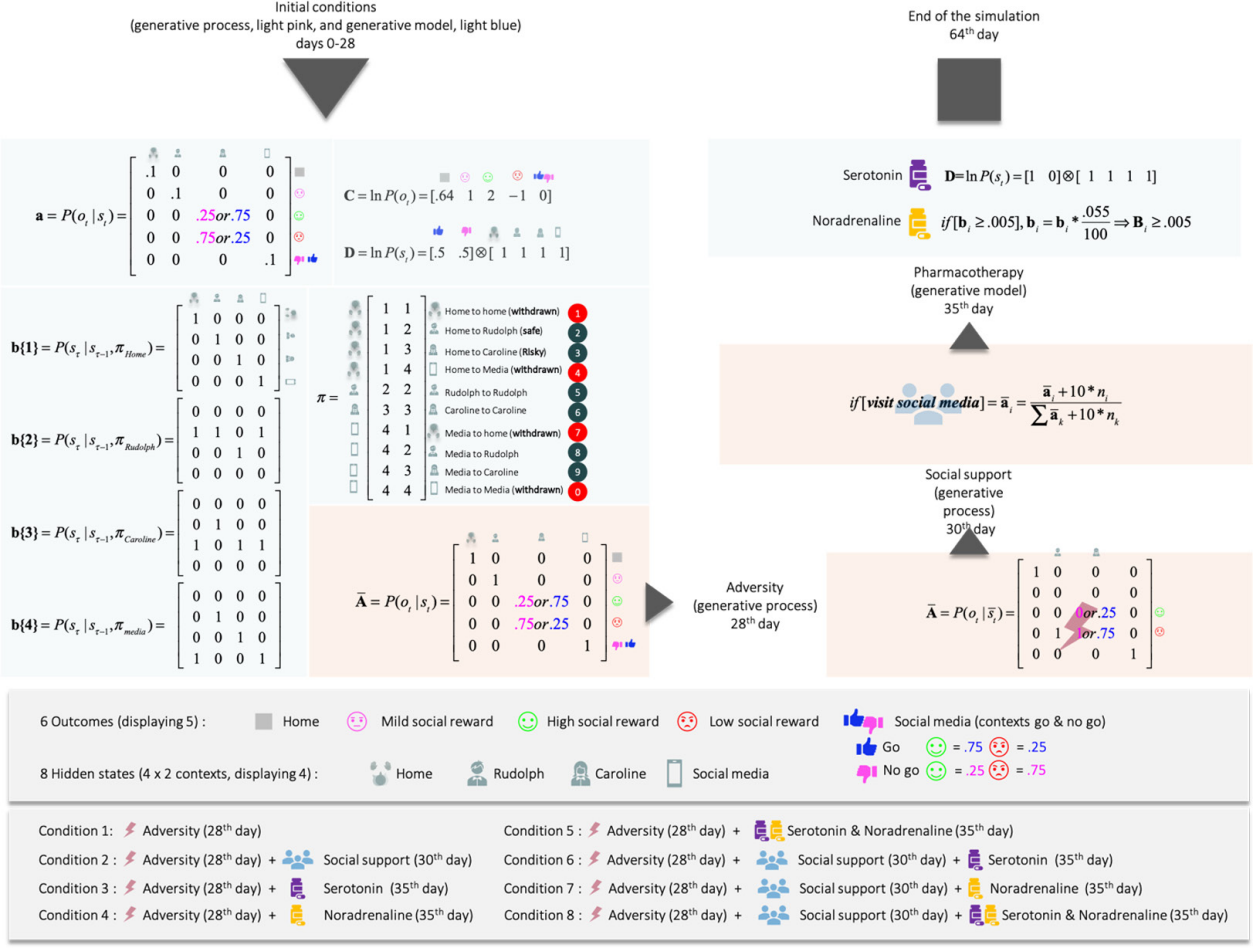
This figure details the likelihood and prior transition probabilities for our generative model of prosocial exchanges. The variables pertaining to the generative model are shown in light blue boxes, while the corresponding parameters of the generative process (i.e., the social world) are shown in light pink. The states and outcomes in this model are generated under two contexts pertaining to Caroline’s availability: available or not available. For ease of visualization, we have shown context-sensitive outcome likelihoods. In other words, there are six potential outcomes, but we have conditioned the epistemic (‘go’ and ‘no-go’) outcome on the context (to generate five outcomes). This simplifies the graphics and is licensed by the fact that only the epistemic outcome is context-sensitive. The top-left section corresponds to the contingencies during the initial exchanges (days 0–28) and corresponds with the narrative description in Figure 1. The adverse life event on the 28^th^ day amounts to Rudolph and Caroline (on a good day) now yielding negative outcomes, and Caroline, even on a good day, affording negative outcomes. Adversity happens when the agent is sensitive to (i.e., prone to learn) the social environment. We implemented this by reinitialising the counts over the sensory prior beliefs of the agent (**a**). Social adversity and support are modelled by changing the precision or reliability of social outcomes in the generative process – in response to social signals. This is a subtle aspect of this model; namely, the generative process or social environment responds adaptively to the agent’s behaviour. As of the 30^th^ day (for the conditions involving social support), we implement social support by adding counts (+10) to the likelihood of the environment counts (+10) for the cells corresponding to the mappings ‘Rudolph and positive outcomes’, ‘Caroline good day context and positive outcome’, and ‘Caroline busy day context and negative outcome’. The n_i corresponds to the number of times the agent visited the location a_i. The increase in counts has the ultimate consequence of driving the probability mapping in the (**A**) of the generative process towards and beyond their initial values more. A ‘+10’ is added to the cells every time the agent solicits the epistemic cue (i.e., social media). This implements the social signalling characteristic of adaptive low mood. Pharmacological interventions on the 35^th^ day include the following: Serotonin provides an optimistic bias by changing prior beliefs about the initial states, in favour of the ‘Go’ context (from .5;.5 to .99;.01). Noradrenaline decreases the precision of the transition probability matrices **B** (i.e., it increases uncertainty about future states), which leads to a gradual accumulation of uncertainty about unvisited states. Through the expected ambiguity component of expected free energy **G**, it tends to motivate exploratory behaviours. The agent continues to learn the state transition after we administer noradrenaline.

	Social withdrawal	Policies that do not lead to an encounter with social partners (see Figure 3) 1: Stay home, stay home (starting point, Figure 1) 4: Stay home, go to social media 7: Go to social media, go back home 10: Go to social media, stay on social media	


### Baseline

**Figure 4:**
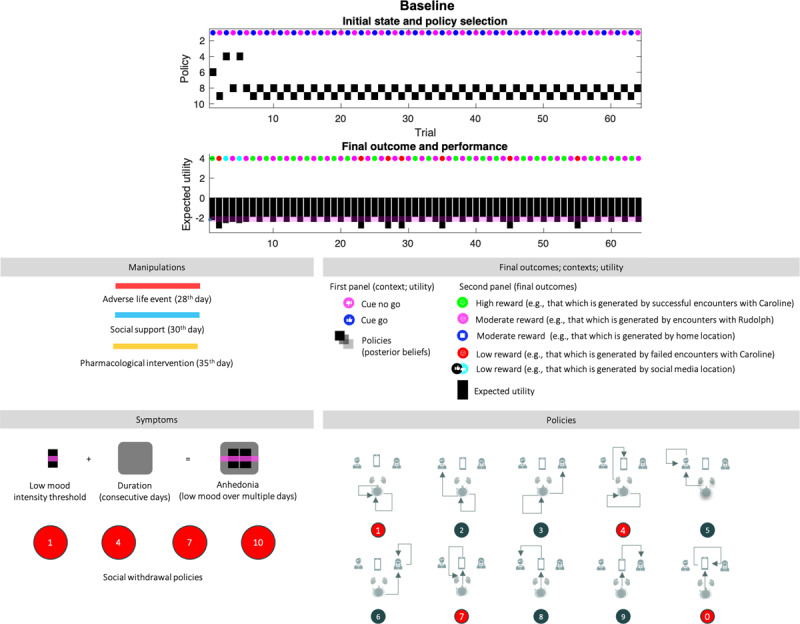
**Baseline. Top panel:** The upper images show the posterior expectation of each of 10 policies (see method, Figure 2) as they evolve from day to day (64 in total). The small circles in the upper part of these panels indicate the observed outcomes (context in the first panel, and outcome in the second). The context changes every other day. The pragmatic value of these outcomes is shown as a (black) bar chart in the second panel. **The lower panel** describes the interventions that depend on the condition, and the symptoms, which are: (i) anhedonia when pragmatic value or reward (black bars) are below the pink bar over multiple days (duration, black shaded rounded rectangles), and (ii) social withdrawal, expressed by policies 1,4,7, and 10. The lower left panel provides a legend (upper) and a graphical description of the policies (lower). The intensity component of anhedonia corresponds inversely to the expected utility of a policy, or the extent to which it will yield preferred outcomes. Narratively speaking, this amounts to expecting socially rewarding outcomes when engaging a certain action. The intensity component of anhedonia is thus defined as low appetitive action. We assume that normal levels of appetitive action correspond to the expected utility experienced on most days, for a healthy (baseline) agent (pink line). The duration component of anhedonia corresponds to the number of consecutive days. A normative assessment of anhedonia thus would involve 14 consecutive days, as is the case in the condition of severe depression below.

**Figure 5:**
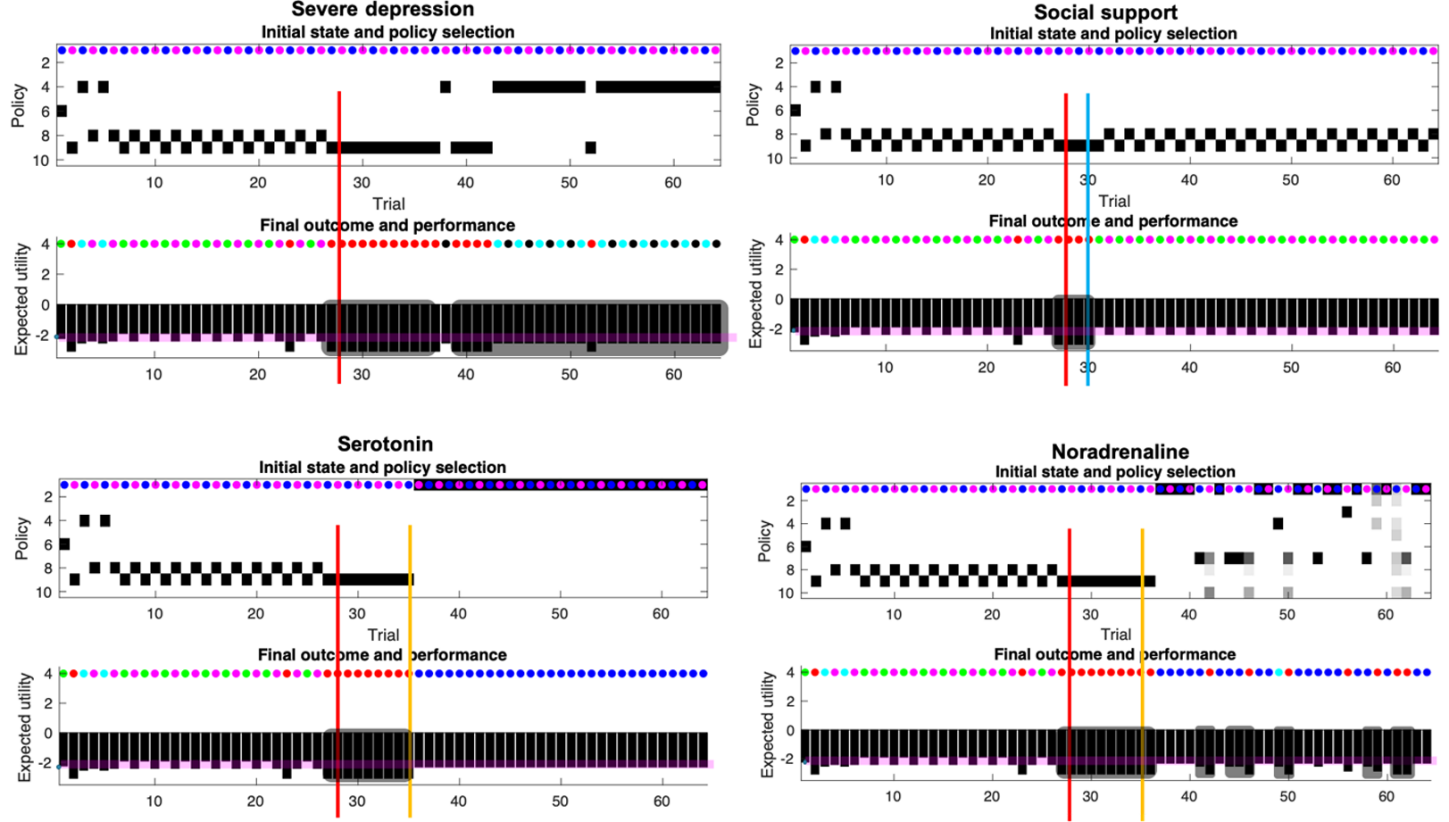
**Responses to intervention.** This figure uses the same format as the upper panels of Figure 4. Interventions are indicated by the solid lines (red line: social adversity; blue line: social support; orange line: pharmacotherapy). The plots report the simulated responses to social adversity (red lines in all quadrants), and the remedial effects of social support (blue line in the second quadrant). Quadrants with orange lines show the corresponding effects of pharmacotherapy (serotonin or noradrenergic). The four treatment conditions show the same behavior over the first 28 days as the baseline scenario. This figure reports the results of conditions 1 to 4.

**Figure 6:**
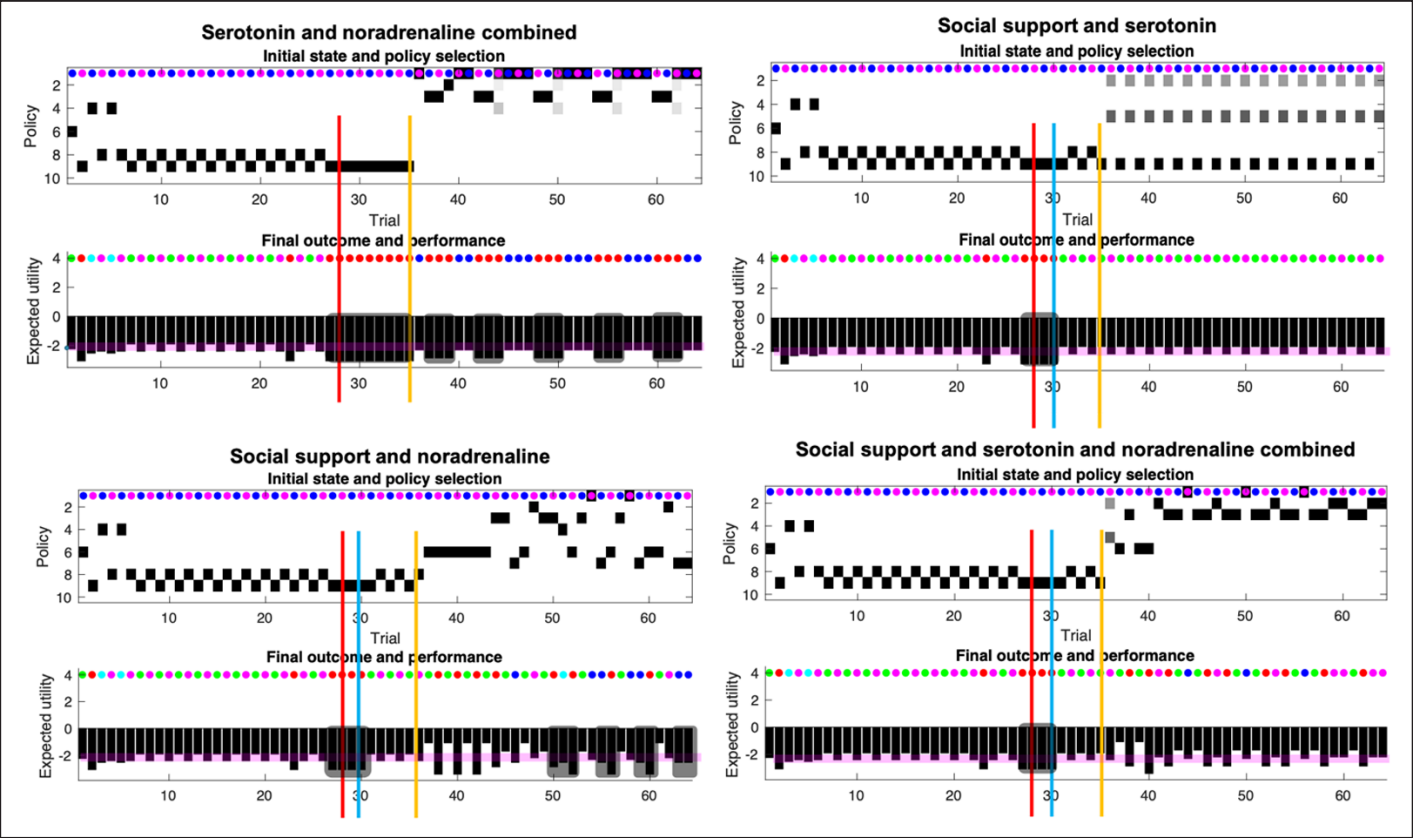
**Responses to pharmacotherapy and social support.** This figure uses the same format as Figure 4 and 5. Here, we report the responses to the final three conditions; namely, responses to serotonin and noradrenaline and combinations of drug treatment (yellow line), after social support (blue line).

**0 Baseline (*Figure 4*):** The first 28 trials are equivalent across all simulations. In the absence of adversity, the agent skillfully responds to contextual changes by shifting between action policies that yield a (risky) high social reward and a (safe) moderate social reward. After the 7^th^ trial, the agent always engages epistemic policies; foraging on social media first, followed by exploitative behaviour resulting in positive or negative outcomes. Before the 7^th^ trial, the agent is still learning her prior beliefs about social partners and figuring out what policy will best suit her preferences; hence the different policies (6,9,8,4, see ***Figure 4***, bottom right for a visual description of each policy). After the 23^rd^ day, the agent misreads the situation: Caroline was having a good day, but the agent perceived a negative outcome (e.g., by misinterpreting Caroline’s behavior during the encounter). The expected utility remains high over all (above the baseline; the pink line), and crucially, there are no consecutive days of anhedonia. ***Figures 5*** and ***6*** use the same format as the upper panel in ***Figure 4*** to show the effects of various interventions on social adversity and support, with or without pharmacological interventions.

### Severe depression, social support, serotonin and noradrenaline

**1 Severe depression (*Figure 5*, upper left quadrant):** The agent experiences social adversity on the 28^th^ trial (i.e., a rejection from Rudolph and Caroline), and has no social support (i.e., her signaling has no effect on Caroline and Rudolph). The adverse life event entails ongoing exposure to negative outcomes. The increase in exposure to negative outcomes is caused by a change in the generative process, which now yields 0% chance of generating a mildly rewarding outcome at the Rudolph state (previously 100% chance), and a 100% chance of generating a negative outcome at that same state. In addition, there is now a 0% chance of a positive outcome and a 100% chance of a negative outcome at the Caroline state during the no go context (busy day), and the probability of Caroline yielding a positive outcome on a good day has been inverted. Now, even on a good day, Caroline only affords a 25% chance of a positive outcome (see adversity on the 28^th^ day, ***Figure 3***). Importantly, the adverse life event affects both the generative process (making bad outcomes more likely for Caroline, and unavoidable for Rudolph), and the generative model by resetting the concentration parameters to their initial values (as they were at trial 1). The motivation for reinitializing the counts is primarily to sensitise our agent to novel outcomes. This sensitisation rests on the fact that learning slows down with the accumulation of concentration parameters (e.g., during the first 27 days). Because the agent’s generative model reverts to its initial settings, the agent expects to obtain positive outcomes at Caroline’s on her good days, for some time after the adverse life event. This explains why our agent keeps selecting policy 9, which leads to Caroline, on multiple days (8 days) after the adverse event.

In our simulation, such a manipulation does not map onto a biological process that we would have aimed to reproduce in silico. It is simply an artefact of the design. Narratively, it may be said that it simulates the awareness of a change in social context caused by a functional forgetting in short-term memory (i.e., from trial 1 through 28) reinstating the agents initial memory parameters (i.e., at trial 1). This leads to an increased sensitivity to the novel social environment. In this particular sense, the resetting of concentration parameters is arguably consistent with the phenotype of depression. Early childhood adversity is a risk factor for depressive disorder by sensitizing the individual to proximal environmental stressors later in life —e.g., making the agent more likely to undergo parameter reset after an adverse life event (Starr et al., 2014) and memory disruptions and negative biases are commonly associated with depression—e.g., acquiring negative bias based on the learning of pessimistic expectations after adverse life events (Dillon & Pizzagalli, 2018). A simulation explicitly aimed at studying the impact of functional forgetting on treatment course could either systematically vary the depth of forgetting or use hierarchical models to allow forgetting to emerge naturally from learning and inference of higher-level contextual states (Hesp et al., 2021).

Occasionally, the agent experiences a negative outcome when Caroline was supposedly having a good day (as indicated by the Go cue). This occurs on average about 25% of the time, because outcomes are generated from the likelihood mapping in ***Figure 3***, which shows there are intrinsic uncertainties in the mapping from Caroline’s mood to positive or negative outcomes (25% chance of failure on Caroline’s good days, 75% chance of failure on Caroline’s bad days). On average, the agent will get a dissatisfying outcome 25% of the time the agent visits Caroline on a good day, because of the constitution of the likelihood mapping (***Figure 3***). The agent is not misinterpreting the cue. It is Caroline that exhibits intrinsic variability.

The agent persistently evinces a low mood, below baseline (i.e., intensity of anhedonia). 14 days after the adverse live event, the agent shifts to a social withdrawal policy (4). This is caused by acquiring a pessimistic likelihood about the outcomes afforded by Caroline and Rudolph. Without intervention, the pessimistic likelihood is successively reinforced.

**2 Social support (*Figure 5*, upper right quadrant):** In this scenario, the agent experiences adversity on day 28 but is provided with social support 2 days later (i.e., her social signaling changes Caroline’s and Rudolph’s behaviour). Following this, the agent’s mood recovers, relative to the baseline condition. This is because Caroline becomes more reliable and the agent is certain that Caroline will show up on a good day, and not on a busy day. This scenario corresponds to what is expected under both the social risk hypothesis and our EST of depression. When the environment is adaptive (i.e., responsive), low mood causes the agent to regain typical functioning – via social signalling. Note that social support failed in simulations where the support was delayed by more than 2 days. After 2 days without support, the pessimistic beliefs become too robust, and no amount of social support is enough to reshape the prior. When the support comes too late, the agent spirals into severe depression. Of course, the critical period of intervention of 2 days depends on the parametrisation of the generative model. Under different parameter values, the critical period could be extended. This speaks to the importance of the timing of social interventions to effectively interrupt and revert the learning of the pessimistic likelihood. More formally, the adaptive response comes from a change in the likelihood of the generative process (see ***Figure 3***), which by generating certain outcomes, leads to a learning of the likelihood matrix. This learning assigns high probabilities to the mappings between the Rudolph state and the high social reward (instead of the moderate social reward), between the Caroline state and the high social reward on the ‘go’ context, and between the Caroline state and the low social reward on the ‘no go’ context. The behavioural manifestation of the social intervention is a return to the correct policy, given the context, namely 8 and 9.

We now consider the therapeutic effects of pharmacotherapy in the absence of social support.

**3 Serotonin (*Figure 5*, lower left quadrant):** Serotonin upregulates prior expectations over the ‘go’ state at the beginning of the trial (**D**). The intervention based solely on serotonin precludes consecutive days of low mood. However, social withdrawal remains (policy 1). Given that the agent receives no social support, the likelihood of receiving negative outcomes from Rudolph on either day is still 100%. The likelihood remains pessimistic after the social adversity on the 28th day; hence the best move for our agent is to stay at home (policy 1), despite the serotonergic bias on beliefs over the go context.

Administration of serotonergic antidepressants induces very strong expectations of Caroline having a ‘good day’, which had the (unintended) side-effect of countering our agent’s epistemic drive. The agent experiences multiple bad outcomes between the moment of the adverse life event and the beginning of the pharmacotherapy, even on good days. The consequence of this is that the expected utility of good days reduces as the agent is left with neither an epistemic nor a pragmatic drive—and opts to stay at home instead. This slightly counterintuitive effect of serotonergic pharmacotherapy underscores the clinical relevance of (1) the timely administration of antidepressants (e.g., before further negative associations become dominant), (2) the support of antidepressants with other types of interventions (i.e., this effect does not occur when combined with social support in our simulations), and (3) the further investigation of potential ways to model and predict the (side-)effects of antidepressants.

**4 Noradrenaline (*Figure 5*, lower right quadrant):** Noradrenaline gradually increases uncertainty about future states (i.e., increases uncertainty in the transition **B** matrices), which underwrites a loss of precise belief-updating during planning—and motivates exploratory behaviours, through the expected ambiguity (in **G**). This is reflected in ***Figure 5*** (lower right quadrant, top panel) showing imprecise beliefs over policies 1 to 10. Noradrenaline intervention engenders several days of low mood after administration, which are generally associated with social withdrawal (policy 7). There is a combination of withdrawal policies (1,4,7), and uncertainty over these policies; e.g., the agent sometimes ends up going to Caroline and receiving a negative outcome (e.g., day 42). Episodes of anhedonia and social withdrawal are short, but present nonetheless, which suggests that the agent is still depressed. We next turn to the effects of combining pharmacotherapy with social support by repeating the above conditions in the setting of a responsive social context.

### Combined interventions

Computationally, social support, serotonin, and noradrenaline operate the same way as described above, whether they are administered individually or combined. What changes are the behavioural effects. To understand these novel effects, we must pay attention to the temporal structure of the depressed system (i.e., the coupled generative model and process). Social support will be the first intervention to impact the generative model (the agent part of the system) by generating the outcome on the basis of which inference and learning operate. Serotonin will act first by influencing initial states (**D**), and finally noradrenaline will act by influencing policy planning (through **B**). The selected policy, if it involves going to the social media state, will influence the probability of outcomes in the generative process (see 30th day, ***Figure 3***), which will then loop back into the generative model to influence inference and learning.

**5 Serotonin and noradrenaline combined (*Figure 6* lower left quadrant):** After the pharmacotherapy on day 35, the agent experiences episodes of anhedonia at regular intervals. However, these are characterised by perceived negative social encounters with Caroline, not social withdrawal. According to the specifications of our simulation, this means that the agent does not meet the requirements for severe depression (i.e., anhedonia and withdrawal criteria). The agent alternates between policies 1, 2, and 3, which do not involve going to social media. This is arguably because serotonin promotes an optimistic bias, meaning that no information foraging is required (e.g., going on social media).

**6 Social support and serotonin (*Figure 6*, lower left quadrant):** In this condition, there is no withdrawal and overall, the mood states are non-depressed (above baseline). This condition combines an optimistic bias with an increase in social stability, yielding high certainty about the reception the agent will receive from Caroline and Rudolph. Since beliefs about policy-dependent state transitions remain the same, there is no need to explore. On Caroline’s good days, the agent approaches Caroline, and on her busy days, the agent engages Rudolph. Note, however, that the agent remains uncertain about which policy to pursue, and compared to the scenario combining noradrenaline, serotonin, and social support, the agent never engages pragmatic policies (e.g., 6).

**7 Social support and noradrenaline (*Figure 6*, lower right quadrant):** This condition yields a variety of responses, and some short episodes of low mood. These are sometimes caused by social withdrawal (e.g., days 64,63), and sometimes by high risk-taking (e.g., day 60), expressed by policy 6. The exploration of the policy space in this scenario is driven by the slow decrease in precision over the transitions (**B** matrices), coupled with an increase in social partners’ reliability.

**8 Social support and serotonin and noradrenaline combined (*Figure 6*, upper right quadrant):** The agent experiences adversity but has social support and access to pharmacotherapy. This scenario largely precludes social withdrawal and consecutive days of anhedonia, and the agent is highly optimistic. Almost on every occasion, the agent engages policy 3 (i.e., wait, then approach Caroline), which explains mood episodes below baseline. Otherwise, the agent engages policy 2 (i.e., wait, then approach Rudolph). Low mood is characterised by risk taking, not social withdrawal. Moreover, for the first time, the agent engages policy 6, which is a pragmatic policy (i.e., going directly to Caroline). This speaks to the effect of noradrenaline, which motivates the agent to disambiguate (future) states that are deemed uncertain, while the few days of withdrawal speaks to the serotonergic bias manifest when Caroline is on a bad day. Note that the epistemic character of a policy concerns the extent to which it disambiguates uncertain transitions. Now, uncertain transitions might be transitions between non epistemic ‘states’, that is, states that provide go/no go outcomes (i.e., if I know where the cue is and where the cue leads, but I do not know if my current location leads to a reward, I will explore this latter transition first, especially if I believe I am in a ‘go’ context, which is what the serotonergic bias does). Hence this condition involves epistemic policies—as in disambiguating behavior—without these policies soliciting epistemic cues.

## Discussion

Using active inference, we have reproduced (artificial) anhedonia and social withdrawal to provide a numerical analysis of the EST of depression. We specified a generative model, involving multiple components that conspired to generate context-sensitive responses to social uncertainty; particularly, the prior preferences for socially rewarding outcomes (e.g., encounters with Caroline). Our results provide support for our hypothesis that depressed mood reflects an adaptive response to interpersonal adversity. Following an adverse life event, our synthetic agent resolved interpersonal uncertainty via social signaling, thereby alleviating her depressed mood. Except for scenarios involving social support, all the conditions we simulated resulted in an above-average duration of episodes of anhedonia and social withdrawal, speaking to unresolved uncertainty. Crucially, we do not claim that depressive psychopathology is adaptive. Indeed, unlike our ‘social support’ condition, the ‘severe depression’ scenario proved to be maladaptive, characterised by unresolvable episodes of low mood and social withdrawal. This may either occur when signalling is defective (e.g., due to personality difficulties, rendering a person unable to deliver the appropriate signals), or when it fails to be received (e.g., cues provided by someone who is socially isolated).

A key aspect of simulations similar to ours is the explicit and formal modelling of the aetiological factors that underwrite the selection of, or inference about, prosocial behaviour; ranging from Bayesian belief updating (i.e., perceptual inference), through to experience-dependent plasticity (i.e., perceptual learning), and to the social and encultured responses of the environment. The active inference framework has an explicit (neuronal) process theory, which could allow future studies to simulate the selective effects of neuromodulatory interventions on the encoding of precision or uncertainty, and its consequences for the agent’s social behaviour. Having a complete model of (aberrant) social inference means that in the future, one could simulate neuronal processes that lend themselves to empirical measurement. Studies along these lines could simulate dopamine responses in order to provide qualitative predictions that could be tested with functional magnetic resonance imaging, e.g.,(D’Ardenne et al., 2008; Schwartenbeck et al., 2015). In this setting, dopamine responses are usually associated with updates to the expected precision of Bayesian beliefs about the policy in play (see Appendix E in Friston, FitzGerald, et al., 2017; Sales et al., 2019)

In our simulations, uncertainty over contingencies decreased every time the agent referred to social media. Narratively, this could be interpreted as the agent signaling (implicitly or explicitly) to Caroline and Rudolph that they should be more consistent in order to provide more support. Note that this does not imply any qualitative change in prosocial responses; it simply corresponds to an increase in the consistency or reliability of responses that may or may not be affiliative. Computationally, this amounts to repairing the environment, such that the prior beliefs of a phenotype are fit for purpose. In other words, the (social) environment changes to match the prior beliefs of its incumbents; thereby reversing the suboptimality implicit in maladaptive depression. Recovery then depends on the sensitivity of the subject’s social environment and on how often she consults social media. Here, the positive effect of combining pharmacotherapy with social support is thought to be attributable to the optimistic bias associated with serotonin (Harmer, 2008; Harmer et al., 2017), coupled with the effect of noradrenaline, which motivates the exploration of states associated with rewards (Aston-Jones & Cohen, 2005).

Given the parametrization of our subject, the best intervention was the combination of social support and serotonin, while the worst outcome—in terms of social withdrawal—was the intervention with serotonin alone (see results table). How these two interventions work together in real participants remains open to question ***Table 3***. Computationally, serotonin provides an optimism bias while social support confirms that bias by returning the social environment to its normal setting (i.e., the setting matches the non-pessimistic expectations of the agent). However, when social support is lacking, serotonin leads to repeated, failed social encounters and social isolation. It is unclear whether serotonergic antidepressants are direct mood enhancers. Rather, it is suggested that antidepressants work by augmenting positive emotional processing, which then has positive effects on other psychological factors (Harmer et al., 2009). Our simulation results highlight this more complex systemic interaction between the psychosocial and neurocognitive aspects of depression and stresses the importance of social support. Indeed, social support in older adults is known to have alleviating, bidirectional effects on symptoms of depression and anxiety. Social disconnectedness appears to predict perceived isolation, which itself predicts higher depressive symptoms, and *vice versa* (Santini et al., 2020). Adolescents who self-report higher perceived social support at age 19 are less likely to show depressive symptoms one year later (Scardera et al., 2020), and reviews emphasise the significant protective effects of perceived emotional and instrumental support, as well as social network diversity in the general population (Santini et al., 2015). The strength of the positive effect of social support, of course, rests on the subject-specific parametrization, which we can expect to vary across real subjects. For instance, we initialized our subjects as ‘blank slates’ with respect to state transitions. However, this would be expected to vary across participants based on their individual experiences and development. This may also vary based on the volatility of the (prosocial) environment prior to the occurrence of social adversity. Again, our proposal is a proof of principle, and is only meant as a general portrait of what is feasible, when considering the social environment in computational phenotyping.

**Table 3:** This table provides a summary of results in terms of the percentages of days post-adversity (out of 36) during which the synthetic subject met the subjective criteria for anhedonia in terms of intensity (expected utility below threshold) and duration (two or more consecutive days) and the behavioural criteria for social withdrawal.


	ANHEDONIA			SOCIAL WITHDRAWAL
	
	INTENSITY CRITERION: EXPECTED UTILITY BELOW 95% CI (% OF 36 DAYS POST-ADVERSITY*)	DURATION CRITERION: 2 OR MORE CONSECUTIVE DAYS (% OF 36 DAYS POST-ADVERSITY*)		BEHAVIOR CRITERION: SELECTED POLICY 1,4,7, OR 1 (% OF 36 DAYS POST-ADVERSITY*)

CONDITION 1 Severe depression	100%	97%		61%

CONDITION 2 Adaptive mood (social support)	6%	3%		0%

CONDITION 3 Serotonin	19%	19%		81%

CONDITION 4 Noradrenaline	53%	53%		75%

CONDITION 5 Serotonin and noradrenaline combined	61%	61%		50%

CONDITION 6 Social support and serotonin combined	6%	0%		0%

CONDITION 7 Social support and noradrenaline combined	44%	31%		22%

CONDITION 8 Social support and serotonin and noradrenaline combined	6%	25%		47%


* Starting the first day after the adverse event (day 29^th^).

Our results speak to the Darwinian models of depression synthesised by the Social Risk Hypothesis (SRH) (cf. ***Box 1***). Following the attachment model, simulated agents – displaying anhedonia and social withdrawal – inhibited social risk-taking under social uncertainty. Following the social competition hypothesis, the adverse life event reduced social uncertainty by producing social withdrawal. Consistent with the resource conservation model, after the adverse event, the agent progressively returned to Caroline, so long as the agent knew exactly when to approach her. From the point of view of the SRH, the explanations of the attachment model, the social competition model, and the resource conservation model are all grounded in the dynamics we simulated. The dynamics we simulated were the increase in social uncertainty leading to behavioural and psychological symptoms that either lead to depression—when the social environment is not responsive—or to the restabilisation of the social network—when the social environment is responsive. The Evolutionary System Theory (EST) of depression, which is the recent neurocomputational reinterpretation of the SRH, frames the adaptive mood dynamics integrated by the SRH as an attunement dynamic between evolutionary adaptive priors (here prior preferences), plastic developmental priors (here B and a likelihood A), and a social environment (here a generative process). These would have been selected to conspire to generate adaptive symptoms of depression in order to trigger social network restabilisation (ex. condition 2); social network stability having been crucial to evolutionary success throughout human history (see ***Box 1***). When the social environment fails to respond to the social signalling represented by depressive symptoms, the behaviourally adaptive pessimistic beliefs that produced this signalling spirals into the maladaptive beliefs characteristic of depressive illness.

## Conclusion: future directions

Simulation studies such as ours can be used to simulate both the symptoms and underlying processes of inference *in silico*. Note, however, that our generative model only had one level. By adding levels to the generative model, as in hierarchical (deep) active inference (Friston, Parr, et al., 2017), one could further finetune these affective dynamics. For instance, one could keep lower-level preferences fixed, reflecting their evolutionary origins, while allowing learning in higher-level preferences to change as a function of life experiences (e.g., learning to prefer Rudolph’s underwhelming calm over Caroline’s extravagance). Furthermore, generative models – of the kind used above – can be fitted to individual and population level clinical data; involving some general-purpose tasks related to a disorder of interest (e.g., social decision-making in depression), thereby yielding a novel avenue for computational phenotyping, prognosis, and diagnostic nosology. The idea here is that clinicians could then predict psychiatric trajectories in specific individuals, when conditioned on different available treatment options. The latter could then be used to generate a prognosis and course of treatment tailored for any client, which we believe is perhaps the most exciting promise of generative modelling in clinical psychiatry.

However, before achieving this, there are many conceptual and technical limitations to overcome, which chiefly relate to the treatment of clinical data using computationally meaningful generative models. Behavioral measurements such as hits and misses and associated social withdrawal can be measured in experimental designs that track behaviour in a decision-making task, with a given narrative (e.g., based on vignettes of real-life scenarios). The challenge lies in fitting individual and environmental initial conditions for both the generative model and generative process (see method, ***Figure 3***). For instance, assuming that preferences are endowed by (encultured) evolution, one should provide a reliable estimate of population-level preferences for social encounters. Then, one should assess the degree of precision of empirical priors and measure the expected utility for each action policy. Crucially, in order to implement the effect of social support, one could also gather and translate information about environmental responsivity. This could be done via task-specific questionnaires (e.g., on a Likert scale, how desirable is an encounter with Rudolph versus Caroline? How reliable do you consider Caroline? Etc.). Alternatively, these questions could be answered by data captured by various technologies. Smartphone-based, passive sensing technologies, which can capture behavioural data (e.g., distances travelled, exercise, sleep, social media activity) and psychological data (e.g., affective tone of text entered), might help in this regard (Sapiro et al., 2019). More generally, the specification of environmental components might be achieved by using various local cultural factors (e.g., cultural norms) regarding the responsivity to idioms of distress; i.e., culturally specific ways of expressing illness experience (Kirmayer & Young, 1998).

In short, to achieve clinical utility, generative models of depression should summarise the client’s neurocognitive disposition to learning as well as her social situation, in terms of the environmental responsivity to her signalling. The role of the clinician, then, would be to map the evolutionary (e.g., adaptive priors), neurocognitive (e.g., empirical priors), and social (e.g., environmental responsivity) portrait of specific clients in terms of a generative (phenotypic) model – a Computational Evolutionary Social assessment of sorts. This opens a novel avenue for research, which attempts to quantify both generative models and processes, by bringing together the expertise of cultural, evolutionary, and computational psychiatrists and psychologists. If such an approach proves reliable – and robust predictions can be made regarding the course of illness experience and optimal treatment options – using computational (social and neurocognitive) phenotyping to improve psychiatric assessment, diagnosis, and tailored interventions might become commonplace.

## Software note

Although the generative model changes from application to application, the belief updates underlying the responses simulated in this paper are generic and can be implemented using standard routines (here spm_MDP_VB_X.m). These routines are available as Matlab code in the SPM academic software: *http://www.fil.ion.ucl.ac.uk/spm/*.

## References

[1] Allen, N. B., & Badcock, P. B. T. (2003). The social risk hypothesis of depressed mood: evolutionary, psychosocial, and neurobiological perspectives. Psychological Bulletin, 129(6), 887–913. DOI: 10.1037/0033-2909.129.6.887 14599287

[2] Allen, N. B., & Badcock, P. B. T. (2006). Darwinian models of depression: a review of evolutionary accounts of mood and mood disorders. Progress in Neuro-Psychopharmacology & Biological Psychiatry, 30(5), 815–826. DOI: 10.1016/j.pnpbp.2006.01.007 16647176

[3] American Psychiatric Association. (2013). Diagnostic and Statistical Manual of Mental Disorders (DSM-5®). American Psychiatric Pub. DOI: 10.1176/appi.books.9780890425596

[4] Aston-Jones, G., & Cohen, J. D. (2005). An integrative theory of locus coeruleus-norepinephrine function: adaptive gain and optimal performance. Annual Review of Neuroscience, 28, 403–450. DOI: 10.1146/annurev.neuro.28.061604.135709 16022602

[5] Badcock, P. B., Davey, C. G., Whittle, S., Allen, N. B., & Friston, K. J. (2017). The Depressed Brain: An Evolutionary Systems Theory. Trends in Cognitive Sciences, 21(3), 182–194. DOI: 10.1016/j.tics.2017.01.005 28161288

[6] Badcock, P. B., Friston, K. J., & Ramstead, M. J. D. (2019). The hierarchically mechanistic mind: A free-energy formulation of the human psyche. Physics of Life Reviews. DOI: 10.1016/j.plrev.2018.10.002 PMC6941235 30704846

[7] Badcock, P. B., Friston, K. J., Ramstead, M. J. D., Ploeger, A., & Hohwy, J. (2019). The hierarchically mechanistic mind: an evolutionary systems theory of the human brain, cognition, and behavior. Cognitive, Affective & Behavioral Neuroscience. DOI: 10.3758/s13415-019-00721-3 PMC6861365 31115833

[8] Bruineberg, J., Rietveld, E., Parr, T., van Maanen, L., & Friston, K. J. (2018). Free-energy minimization in joint agent-environment systems: a niche construction perspective. Journal of Theoretical Biology. DOI: 10.1016/j.jtbi.2018.07.002 PMC6117456 30012517

[9] Buckner, J. D., Joiner, T. E., Jr, Pettit, J. W., Lewinsohn, P. M., & Schmidt, N. B. (2008). Implications of the DSM’s emphasis on sadness and anhedonia in major depressive disorder. Psychiatry Research, 159(1–2), 25–30. DOI: 10.1016/j.psychres.2007.05.010 18334272 PMC3688280

[10] Constant, A., Ramstead, M. J. D., Veissière, S. P. L., Campbell, J. O., & Friston, K. J. (2018). A variational approach to niche construction. Journal of the Royal Society, Interface/the Royal Society, 15(141). DOI: 10.1098/rsif.2017.0685 PMC5938575 29643221

[11] Corlett, P. R., & Fletcher, P. C. (2014). Computational psychiatry: a Rosetta Stone linking the brain to mental illness. The Lancet. Psychiatry, 1(5), 399–402. DOI: 10.1016/S2215-0366(14)70298-6 26361002

[12] Cullen, M., Davey, B., Friston, K. J., & Moran, R. J. (2018). Active Inference in OpenAI Gym: A Paradigm for Computational Investigations Into Psychiatric Illness. Biological Psychiatry. Cognitive Neuroscience and Neuroimaging, 3(9), 809–818. DOI: 10.1016/j.bpsc.2018.06.010 30082215

[13] D’Ardenne, K., McClure, S. M., Nystrom, L. E., & Cohen, J. D. (2008). BOLD responses reflecting dopaminergic signals in the human ventral tegmental area. Science, 319(5867), 1264–1267. DOI: 10.1126/science.1150605 18309087

[14] Dillon, D. G., & Pizzagalli, D. A. (2018). Mechanisms of Memory Disruption in Depression. Trends in Neurosciences, 41(3), 137–149. DOI: 10.1016/j.tins.2017.12.006 29331265 PMC5835184

[15] Friston, K. J. (2010). The free-energy principle: a unified brain theory? Nature Reviews. Neuroscience, 11(2), 127–138. DOI: 10.1038/nrn2787 20068583

[16] Friston, K. J., FitzGerald, T., Rigoli, F., Schwartenbeck, P., O Doherty, J., & Pezzulo, G. (2016). Active inference and learning. Neuroscience and Biobehavioral Reviews, 68, 862–879. DOI: 10.1016/j.neubiorev.2016.06.022 27375276 PMC5167251

[17] Friston, K. J., FitzGerald, T., Rigoli, F., Schwartenbeck, P., & Pezzulo, G. (2017). Active inference: a process theory. Neural Computation, 29(1), 1–49. DOI: 10.1162/NECO_a_00912 27870614

[18] Friston, K. J., Parr, T., & de Vries, B. (2017). The graphical brain: Belief propagation and active inference. Network Neuroscience, 1(4), 381–414. DOI: 10.1162/NETN_a_00018 29417960 PMC5798592

[19] Gilbert, P. (1997). The evolution of social attractiveness and its role in shame, humiliation, guilt and therapy. The British Journal of Medical Psychology, 70(Pt 2), 113–147. DOI: 10.1111/j.2044-8341.1997.tb01893.x 9210990

[20] Harmer, C. J. (2008). Serotonin and emotional processing: does it help explain antidepressant drug action? Neuropharmacology, 55(6), 1023–1028. DOI: 10.1016/j.neuropharm.2008.06.036 18634807

[21] Harmer, C. J., Duman, R. S., & Cowen, P. J. (2017). How do antidepressants work? New perspectives for refining future treatment approaches. The Lancet. Psychiatry, 4(5), 409–418. DOI: 10.1016/S2215-0366(17)30015-9 28153641 PMC5410405

[22] Harmer, C. J., Goodwin, G. M., & Cowen, P. J. (2009). Why do antidepressants take so long to work? A cognitive neuropsychological model of antidepressant drug action. The British Journal of Psychiatry: The Journal of Mental Science, 195(2), 102–108. DOI: 10.1192/bjp.bp.108.051193 19648538

[23] Hesp, C., Smith, R., Parr, T., Allen, M., Friston, K. J., & Ramstead, M. J. D. (2020). Deeply Felt Affect: The Emergence of Valence in Deep Active Inference. Neural Computation, 1–49. DOI: 10.31234/osf.io/62pfd PMC8594962 33253028

[24] Hindash, A. H. C., & Amir, N. (2012). Negative Interpretation Bias in Individuals with Depressive Symptoms. Cognitive Therapy and Research, 36(5), 502–511. DOI: 10.1007/s10608-011-9397-4

[25] Hrdy, S. B. (2011). Mothers and others. Harvard University Press.

[26] Huys, Q. J. M., Daw, N. D., & Dayan, P. (2015). Depression: a decision-theoretic analysis. Annual Review of Neuroscience, 38, 1–23. DOI: 10.1146/annurev-neuro-071714-033928 25705929

[27] Huys, Q. J. M., Guitart-Masip, M., Dolan, R. J., & Dayan, P. (2015). Decision-Theoretic Psychiatry. Clinical Psychological Science, 3(3), 400–421. DOI: 10.1177/2167702614562040

[28] Ingram, R. E., Miranda, J., & Segal, Z. V. (1998). Cognitive vulnerability to depression. Guilford Press.

[29] Kaplan, R., & Friston, K. J. (2018). Planning and navigation as active inference. Biological Cybernetics. DOI: 10.1007/s00422-018-0753-2 PMC6060791 29572721

[30] Kirmayer, L. J., & Young, A. (1998). Culture and somatization: clinical, epidemiological, and ethnographic perspectives. Psychosomatic Medicine, 60(4), 420–430. DOI: 10.1097/00006842-199807000-00006 9710287

[31] Klinger, E. (1975). Consequences of commitment to and disengagement from incentives. Psychological Review, 82(1), 1. DOI: 10.1037/h0076171

[32] Montague, P. R., Dolan, R. J., Friston, K. J., & Dayan, P. (2012). Computational psychiatry. Trends in Cognitive Sciences, 16(1), 72–80. DOI: 10.1016/j.tics.2011.11.018 22177032 PMC3556822

[33] Nesse, R. M. (1990). Evolutionary explanations of emotions. Human Nature, 1(3), 261–289. DOI: 10.1007/BF02733986 24222085

[34] Nesse, R. M. (2000). Is depression an adaptation? Archives of General Psychiatry, 57(1), 14–20. DOI: 10.1001/archpsyc.57.1.14 10632228

[35] Nettle, D. (2004). Evolutionary origins of depression: a review and reformulation. Journal of Affective Disorders, 81(2), 91–102. DOI: 10.1016/j.jad.2003.08.009 15306134

[36] Parr, T., & Friston, K. J. (2017). Uncertainty, epistemics and active inference. Journal of the Royal Society, Interface / the Royal Society, 14(136). DOI: 10.1098/rsif.2017.0376 PMC5721148 29167370

[37] Parr, T., Rees, G., & Friston, K. J. (2018). Computational Neuropsychology and Bayesian Inference. Frontiers in Human Neuroscience, 12, 61. DOI: 10.3389/fnhum.2018.00061 29527157 PMC5829460

[38] Price, J. (1967). THE DOMINANCE HIERARCHY AND THE EVOLUTION OF MENTAL ILLNESS. The Lancet, 290(7509), 243–246. DOI: 10.1016/S0140-6736(67)92306-9

[39] Rude, S. S., Valdez, C. R., Odom, S., & Ebrahimi, A. (2003). Negative Cognitive Biases Predict Subsequent Depression. Cognitive Therapy and Research, 27(4), 415–429. DOI: 10.1023/A:1025472413805

[40] Sales, A. C., Friston, K. J., Jones, M. W., Pickering, A. E., & Moran, R. J. (2019). Locus Coeruleus tracking of prediction errors optimises cognitive flexibility: An Active Inference model. PLoS Computational Biology, 15(1), e1006267. DOI: 10.1371/journal.pcbi.1006267 30608922 PMC6334975

[41] Santini, Z. I., Jose, P. E., York Cornwell, E., Koyanagi, A., Nielsen, L., Hinrichsen, C., Meilstrup, C., Madsen, K. R., & Koushede, V. (2020). Social disconnectedness, perceived isolation, and symptoms of depression and anxiety among older Americans (NSHAP): a longitudinal mediation analysis. The Lancet. Public Health, 5(1), e62–e70. DOI: 10.1016/S2468-2667(19)30230-0 31910981

[42] Santini, Z. I., Koyanagi, A., Tyrovolas, S., Mason, C., & Haro, J. M. (2015). The association between social relationships and depression: a systematic review. Journal of Affective Disorders, 175, 53–65. DOI: 10.1016/j.jad.2014.12.049 25594512

[43] Sapiro, G., Hashemi, J., & Dawson, G. (2019). Computer vision and behavioral phenotyping: an autism case study. Current Opinion in Biomedical Engineering, 9(C). DOI: 10.1016/j.cobme.2018.12.002 PMC10544819 37786644

[44] Scardera, S., Perret, L. C., Ouellet-Morin, I., Gariépy, G., Juster, R.-P., Boivin, M., Turecki, G., Tremblay, R. E., Côté, S., & Geoffroy, M.-C. (2020). Association of Social Support During Adolescence With Depression, Anxiety, and Suicidal Ideation in Young Adults. JAMA Network Open, 3(12), e2027491. DOI: 10.1001/jamanetworkopen.2020.27491 33275154 PMC7718598

[45] Schwartenbeck, P., FitzGerald, T. H. B., Mathys, C., Dolan, R., & Friston, K. (2015). The Dopaminergic Midbrain Encodes the Expected Certainty about Desired Outcomes. Cerebral Cortex, 25(10), 3434–3445. DOI: 10.1093/cercor/bhu159 25056572 PMC4585497

[46] Schwartenbeck, P., & Friston, K. (2016). Computational Phenotyping in Psychiatry: A Worked Example. eNeuro, 3(4). DOI: 10.1523/ENEURO.0049-16.2016 PMC4969668 27517087

[47] Schwartenbeck, P., Passecker, J., Hauser, T. U., FitzGerald, T. H., Kronbichler, M., & Friston, K. J. (2019). Computational mechanisms of curiosity and goal-directed exploration. eLife, 8. DOI: 10.7554/eLife.41703 PMC6510535 31074743

[48] Starr, L. R., Hammen, C., Conway, C. C., Raposa, E., & Brennan, P. A. (2014). Sensitizing effect of early adversity on depressive reactions to later proximal stress: Moderation by polymorphisms in serotonin transporter and corticotropin releasing hormone receptor genes in a 20-year longitudinal study. In Development and Psychopathology (Vol. 26, Issue 4pt2, pp. 1241–1254). DOI: 10.1017/S0954579414000996 25422958 PMC5546887

